# Quantitative Trait Loci (QTLs) Associated with Microspore Culture in *Raphanus sativus* L. (Radish)

**DOI:** 10.3390/genes11030337

**Published:** 2020-03-21

**Authors:** Kyeongmin Kim, Yuna Kang, Sol-Ji Lee, Se-Hyun Choi, Dong-Hyun Jeon, Min-Young Park, Suhyoung Park, Yong Pyo Lim, Changsoo Kim

**Affiliations:** 1 Department of Crop Science, College of Agricultural and Life Sciences, Chungnam National University, Daejeon 34134, Korea; katty1502@cnu.ac.kr (K.K.); dkwl3120@cnu.ac.kr (Y.K.); solji2m@naver.com (S.-J.L.); chltpgus1996@naver.com (S.-H.C.); jemdong@cnu.ac.kr (D.-H.J.); 2 National Institute of Horticultural & Herbal Science, Rural Development Administration (RDA), Wanju 55365, Korea; endrmfdl99@naver.com (M.-Y.P.); psh@korea.kr (S.P.); 3 Department of Horticultural Science, College of Agricultural and Life Sciences, Chungnam National University, Daejeon 34134, Korea; yplim@cnu.ac.kr; 4 Department of Smart Agriculture Systems, College of Agricultural and Life Sciences, Chungnam National University, Daejeon 34134, Korea

**Keywords:** radish, microspore culture, regeneration rate, outcrossing, two-way pseudo-testcross model

## Abstract

The radish is a highly self-incompatible plant, and consequently it is difficult to produce homozygous lines. Bud pollination in cross-fertilization plants should be done by opening immature pollen and attaching pollen to mature flowers. It accordingly takes a lot of time and effort to develop lines with fixed alleles. In the current study, a haploid breeding method has been applied to obtain homozygous plants in a short period of time by doubling chromosomes through the induction of a plant body in the haploid cells, in order to shorten the time to breed inbred lines. We constructed genetic maps with an F1 population derived by crossing parents that show a superior and inferior ability to regenerate microspores, respectively. Genetic maps were constructed from the maternal and parental maps, separately, using the two-way pseudo-testcross model. The phenotype of the regeneration rate was examined by microspore cultures and a quantitative trait loci (QTL) analysis was performed based on the regeneration rate. From the results of the culture of microspores in the F1 population, more than half of the group did not regenerate, and only a few showed a high regeneration rate. A total of five significant QTLs were detected in the F1 population, and five candidate genes were found based on the results. These candidate genes are divided into two classes, and appear to be related to either PRC2 subunits or auxin synthesis.

## 1. Introduction

With the recent development of life science technology, the importance of molecular breeding technology as well as hybridization and selection breeding is growing rapidly in seed production. In addition, it is necessary to establish a breeding system to maintain international competitiveness in the vegetable seed market by shortening the development time of new varieties through molecular breeding. However, there is a lack of biotechnology-based varieties due to the difficulty in marker development and insufficient information on genetic resources. The embryo development of flowering plants using microspores is a fascinating system in that it can dramatically promote the process of plant breeding. 

The Brassicaceae includes important plants, such as *Arabidopsis thaliana* L., *Brassica napus* L., *Brassica carinata* L., *Brassica juncea* L., and *Brassica rapa* L. These plants are being used as model genomes for molecular biology research of Brassicaceae. In particular, comparative genomic studies of *Arabidopsis* and cabbage have been actively conducted, and many research papers on cabbage, radishes, and rapeseed have recently been published [1,2,3]. In the Brassicaceae, the radish has been relatively understudied on the genomics level, and research has focused on traits related to F1 breeding, including male sterility. Genomics research on radishes is currently conducted mainly in Korea, China, Japan, and the United States. The genome size of radishes is known to be approximately 530–573 Mb [4,5]. Recently, 402 Mb genome sequences of the Japanese cultivar ‘Aokubi’ have been published. A total of 1345 scaffolds were assigned to the linkage map, spanning 116.0 Mb. However, the sequences are draft scaffolds with a small size of N50 (46.3 kb) and only 116 Mb of these sequences been assigned to chromosomes showing incomplete genome assembly [6]. A continuum-level genome draft has been reported for the North American wild radish in the United States [3]. The assembly size is 254 Mb and N50 is 10.1 kb. In Korea, the assembly of pseudomolecule-level assemblies, rather than contigs or scaffolds, has been announced, which is assembled at 426.2 Mb and the size of scaffold N50 is 1.22 Mb. The scaffold mapped to the chromosome in the standard dielectric is 344 Mb, forming nine pseudomolecules [7]. This shows the level of maturity of the recent *B. rapa* and *B. oleracea* standards. The first genetic maps in radish were made using populations created by cross-breeding between *R. sativus* and *R. raphanustrum* to enhance DNA polymorphism, and the RFLP molecular markers identified in *Brassica* spp. were used. Over time, genetic mapping has been done using various types of markers through intraspecific hybridization. 

Despite the diversity and importance of radishes, little genetic information has been released about this plant. Most horticultural or vegetable crops have relatively long breeding cycles, from six months to a year or more [8]. Conventionally, only one generation per year of radishes is produced. Research on molecular markers related to shortening the breeding cycle is still ongoing, and the introduction of a breeding system using modern breeding technologies based on molecular markers or genomic information should be developed. For radish breeding and genetic research, it is important to shorten the breeding cycle and then create pure lines in a short time frame. Owing to the high self-incompatibility of the radish, it is necessary to secure homozygous genetic resources with fixed traits for the development of gene functional molecular markers [9]. Because the brassica crops have pronounced self-incompatibility, pollination is difficult [10,11]. For this reason, bud pollination, the process of opening immature pots, and attaching flower pots of mature flowers are utilized [12,13]. In addition, it requires a lot of time and effort to develop fixed lines since at least it takes 6–7 generations of single seed descent. In order to solve this problem, a haploid breeding method to obtain a homozygous plant in a short period of time by doubling chromosomes through the induction of a plant from haploid cells has attracted attention [14,15]. Haploids can quickly produce homozygotes through chromosomal duplications, which can significantly shorten the breeding age through self-pollination [16]. Haploid plants are also used in many fields, as they are advantageous for screening phenotypes of superior recessive traits that are not expressed in diploids, as well as for use in interspecific cross-breeding. Haploid is a general term describing sporophytes with a single set of parental chromosomes. Normal haploids are poorly grown and have low values due to difficulties such as infertility, but the embryoid body grown by doubled haploid lines are characterized by high purity. 

In traditional breeding, the selection of these quantitative traits is usually performed by phenotypic testing, mainly by the grower’s experience, but these traits are easily influenced by the external environment. Therefore, by creating a genetic map of quantitative trait loci (QTL) with molecular markers that are closely related to the target trait, marker-assisted selection (MAS) can be used to simplify the breeding process and improve efficiency. These molecular markers have become an essential tool for plant genetics and breeding studies. Starting with RFLP (Restriction Fragment Length Polymorphism) markers, various labels, such as RAPD (Random Amplification of Polymorphic DNA), SSR (Simple Sequence Repeat), and SNP (Single Nucleotide Polymorphism), have been developed. Among them, SNPs have been developed. They are molecular markers that are suitable for genome analysis, mapping, and marker-assisted breeding.

In this study, to improve the molecular breeding efficiency of radishes, we generated linkage maps with an F1 population and performed a QTL study associated with the regeneration rate by microspore culture, which is considered to be important in radish. Using an F1 population derived from an intra-specific cross between the high regeneration rate radish lines and low regeneration rate radish lines, ‘GX71’ and ‘GX50’, respectively, genetic linkage maps were constructed with SNPs. In order to obtain a large amount of SNP markers, low-cost and high-efficiency genotype-by-sequencing (GBS) was performed, and the SNP markers were obtained by filtering several steps to secure reliable associations between genotypes and phenotypes. The results will be utilized to develop molecular markers associated with the efficiency of microspore culture so that early stage screening is possible for the doubled haploid breeding programs in radishes.

## 2. Materials and Methods 

### 2.1. Plant Materials and Genomic DNA Extraction

A population of 62 F1 individuals was derived from a cross of ‘GX50’ × ‘GX71’, which were maintained in the National Institute of Horticultural and Herbal Science, Rural Development Administration, Wanju, Korea. The F1 mapping population consists of 62 progenies from an inter-specific cross between two heterozygous genotypes.

The total genomic DNA of each parent and the progenies were extracted from young and fresh leaf tissues using the modified cetyltrimethylammonium bromide (CTAB) method [17]. The collected leaves were immediately stored in liquid nitrogen, and then transferred to the laboratory and stored at −80 °C until use. The extracted DNA samples were diluted to 20 ng/µL and then used for library preparation. DNA concentration and purity were determined using a Nano stick-S (Scinco, Seoul, Korea).

### 2.2. Microspore Culture and Regeneration

Microspore cultures were conducted according to Na et al. [18] with some modifications. To isolate the microspores, 30 flower buds of 2–3 mm size before flowering were selected and collected in gauze, and then surface sterilized with 1% sodium hypochlorite (NaOCl) for 15 min on a shaker followed by three rounds of 3-minute washing with sterile water. The surface-sterilized buds were then ground in mortar and pestles in 2–3 mL of NLN [19,20] medium supplemented with 13% sucrose, filtered through a 45-µm sieve, and suspended in 30 mL of NLN medium. Centrifugation was performed at 1000 rpm for 3 min, and when the centrifugation was completed, the supernatant was discarded in a clean bench. The same process was repeated three times by adding a new NLN medium. The separated microspores were suspended in 75 mL of NLN medium containing activated carbon at a density of 40,000 microspores to 1 mL and then divided into 2.5 mL petri dishes (60 mm × 15 mm) for incubation and sealed with parafilm and wraps. On average, the concentration and the volume were adjusted to include one flower bud per petri dish [21]. To the induce embryonic development of the microspores, they were treated for 48 hours in a dark condition of 30 °C and then maintained at 25 °C for about 2 weeks. Afterwards, the embryos were transferred to a light condition of 25 °C and shaken at 75–80 rpm to observe embryogenesis. Microspore-derived embryos were maintained in a shaker incubator up to about 7 mm in size and transferred to a half strength of Murashige-Skoog (MS) [22] solid medium containing 3% sucrose for plant regeneration. These counted embryos were defined as embryos from microspores collected at an early stage. The regeneration rate was calculated using the equation below.

Regeneration rate (%) = [The total number of induced embryos from microspores culture/30(mL) × 100,000 (microspores/mL)] × 100

### 2.3. Genotyping-by-Sequencing (GBS) Library and Illumina Sequencing

A sequencing library was prepared according to the GBS protocol in [23] using two restriction endonucleases, *Nsi*l-HF and *MseI*, which were successfully used for constructing GBS libraries in our previous report [24]. For the construction of the GBS library, a set of 64 barcoded adapters was generated from complementary oligonucleotides with the *Nsi*l-HF (New England Biolabs, Ipswich, MA) overhang sequence and unique barcode of length 5–10 bp and they have been tested using two parental lines to determine the optimum conditions for the GBS analysis. The adapter was treated with 2000 U T4 DNA ligase (New England Biolabs, Ipswich, MA) for 2 h at 22 °C and maintained at 65 °C for 20 min to eliminate ligase activity. Barcoded DNA samples were collected into a 50 mL falcon tube and purified using a PCI (phenol-chloroform-isoamyl alcohol) extraction method. It was then size-selected with AMPure XP beads (Beckman Coulter, High Wycombe, UK) to exclude small fragments which are preferentially amplified by polymerase chain reaction (PCR). The product from PCR went through size selection again to be the final product for further processes. The concentrations of the GBS libraries were determined using a Qubit fluorimeter (Thermo Fisher Scientific, Grand Island, NY, USA). Subsequent sequencing was performed on an Illumina HiSeq 2500 platform. The sequencing data are deposited in the NCBI’s SRA database (PRJNA610719).

### 2.4. Sequence Data Analysis and SNP Calling

**Figure 1:**
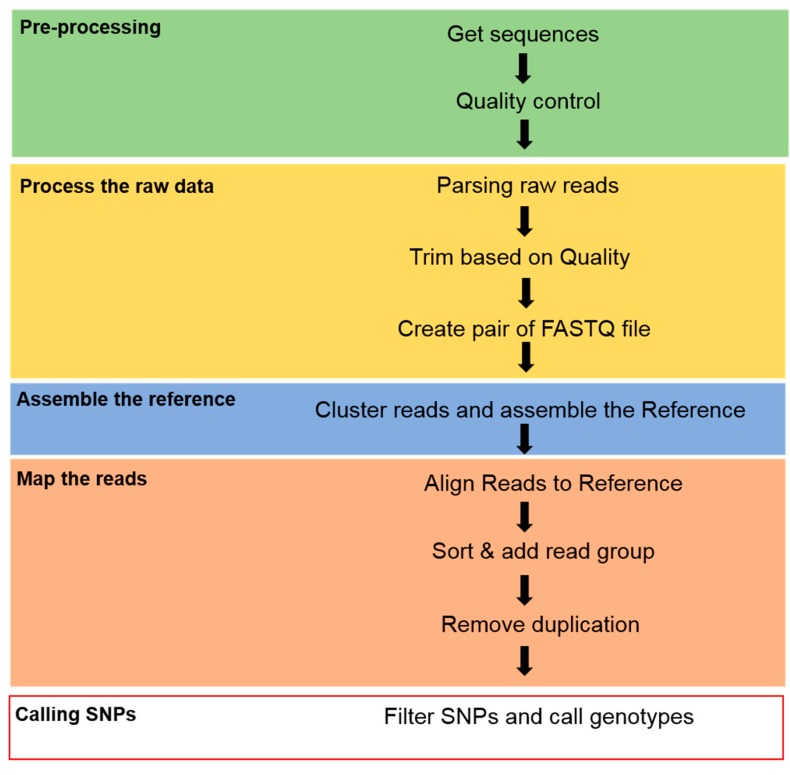
A pipeline for calling single nucleotide polymorphisms (SNPs) from genotype-by-sequencing (GBS) data.

When this study was completed, there was a reference genome available for aligning Illumina raw reads for SNP discovery. Raw reads were de-multiplexed and the barcode sequences were removed. Any sequences not containing the expected restriction sites for both enzymes were removed. Subsequently, the reads were filtered and trimmed using recommended settings in Trimmomatic-0.39 [25]. Burrows–Wheeler Aligner (BWA) software [26] was then used to assemble and to align the clean reads from each individual against the radish’s reference genome. Genomic data (Rs 1.0 chromosome) at the chromosome pseudomolecule level of the Radish Genome Database (http://radish-genome.org/) were used as the reference genome [27]. Alignment files ware converted to bam files using the SAMtools software [28]. A genome Analysis Toolkit (GATK) and Picard tools [29] were used for variant calling. The GATK ‘HaplotypeCaller’ was used to find all possible SNP and indel sites. In addition, filtering was performed using GATK ‘VariantFiltration’ (Figure 1).

### 2.5. Linkage Map Construction

The F1 individuals were genotyped based on marker polymorphisms. The marker-segregation data were analyzed with JOINMAP Version 4.1 by treating the segregation data of the markers as a “cross pollination” (CP) population. The significance of each allele was tested by JoinMap, which further filters for independence of segregation using logarithm of odds (LOD) scores [30]. The significance was determined at a LOD threshold of 5.0 and 8.0. SNP markers, which were heterozygous in only one of the parents (testcross markers), were scored either lm × ll or nn × np depending on the parent, and heterozygous markers in both parents with two alleles (intercross markers) were scored as hk × hk. The segregation patterns lm × ll and hk × hk were used for the maternal map construction, while the patterns nn x np and hk × hk were used for the paternal map according to the two-way pseudo-testcross mapping strategy [31]. Chi-square tests were performed to test for deviation from the expected Mendelian segregation ratio for each marker. The testcross markers were tested against a Mendelian segregation ratio of 1:1 using a chi-square test (*P* < 0.05), while those intercross markers were tested against a 1:2:1 ratio (*P* < 0.05). SNP markers with more than 30% missing data were removed from the analysis. After linkage groups (LGs) were computed, their number was assigned according to the chromosome number of the mapped marker. The regression algorithm and Kosambi [32] mapping function were used in the marker distance calculation, expressed in centiMorgans (cM). Maps were viewed using MapChart 2.3 [33].

### 2.6. QTL Analysis

Inclusive composite interval mapping (ICIM) (http://www.isbreeding.net/) software was used to analyze the LOD profiles with informative markers, as detected by Joinmap. The LOD thresholds for QTL significance were determined by a permutation test (1000 replications) with a genome-wide significance level α = 0.1. ICIM tests both additive and epistasis effects and it avoids the possible increase in sampling variance and the complicated background marker selection process, which are common problems in composite interval mapping.

## 3. Results

### 3.1. GBS Library Sequencing and SNP Calling

The Hiseq 2500 platform was used to conduct the paired-end (100 bp each) sequencing of the GBS library for the parents and 62 progenies. A total of 422,282,974 reads and 42,650,580,374 bases (42.65 Gb) were generated. A total of 97,772 SNPs and InDels were detected using the GATK’s VariantsToTable command. Among these, only 80,283 SNPs were called, and then 22,158 SNPs that have all of the parental genotypes were selected again. SNPs were evenly distributed throughout the genome.

### 3.2. Linkage Map

**Figure 2:**
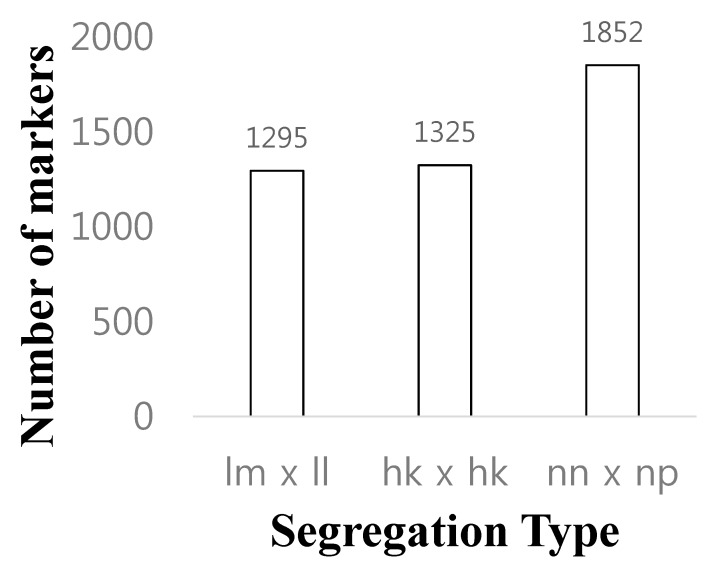
Segregation types of polymorphic SNP markers. The *x-axis* indicates the three segregation types; the *y-axis* indicates the number of markers included in each marker type. lm × ll = ‘GX50’ heterozygous ‘GX71’ homozygous. hk × hk = ‘GX50’ heterozygous ‘GX71’ heterozygous. nn × np = ‘GX50’ homozygous ‘GX71’ heterozygous.

**Figure 3:**
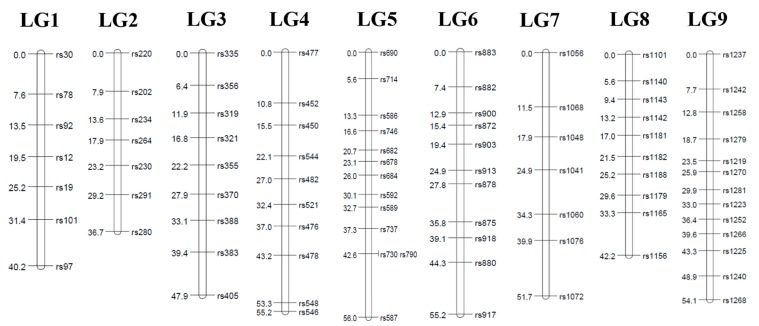
Genetic linkage map of GX50 constructed from an outbred population between GX50 and GX71. The map was constructed from 87 discovered SNPs and genotyped using GBS, including SNPs from all three pseudo-testcross segregation types.

**Figure 4:**
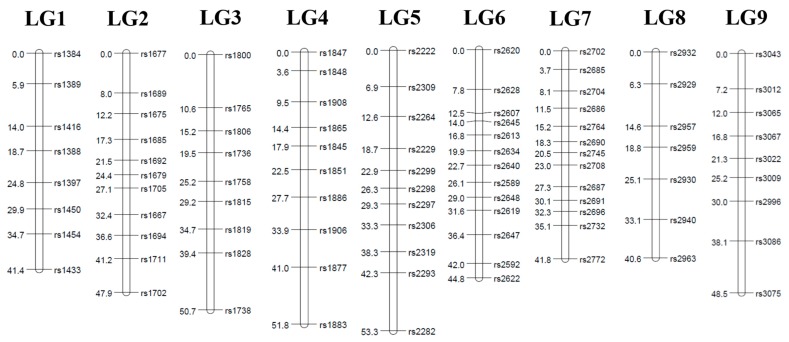
Genetic linkage map of GX71 constructed from an outbred population between GX50 and GX71. The map was constructed from 91 SNPs discovered and genotyped using GBS, including SNPs from all three pseudo-testcross segregation types

A linkage analysis was done in the F1 mapping population consisting of 62 individuals from a cross ‘GX71’ (high regeneration rate) between ‘GX50’ (low regeneration rate). Two individuals were excluded from the analyses because they have too many missing genotypes. We keep the missing rate below 30% so that the linkage analysis is properly performed. As a result, a total of 4462 SNPs were selected by first classifying genotypes into lm × ll, nn × np, and hk × hk types (see the Section 2.5 for details). Finally, based on the genotypes of the parents, monomorphic markers were removed. Consequently, the total number of SNPs for genetic mapping was chosen as follows: 1295, 1325, and 1852 segregating SNPs with the classes of lm × ll, hk × hk, and nn × np, respectively (Figure 2). Markers deviating significantly (*P* < 0.05) from the Mendelian segregation ratio for dominant and codominant markers, respectively, were excluded from the analysis. To increase the mapping efficiency, locus pairs or locus groups with identical genotypes were identified and a single marker was selected to represent the group. After removing the redundant SNP markers, the final set of 178 SNPs remained for linkage map construction. The linkage maps were constructed using Joinmap 4.1 software (logarithm of odds (LOD) ≥5). The linkage maps contained nine linkage groups, which were consistent with the basal chromosome number of the radish. Because of the lack of bi-parental marker types that can connect parental genetic maps in this study, we first identified a set of SNP markers to assign the nine radish chromosomes to nine LGs. For the maternal linkage map, 87 markers were mapped from all three pseudo-testcross segregation types (Table 1, Figure 3). The length of the maternal linkage map was 439.2 cM, with the longest linkage group, LG5 (56.0 cM), and the shortest linkage group, LG2 (36.7 cM). The average interval marker distance was 5.9 cM and the maximum gap size ranged from 7.7 cM (LG9) to 13.4 cM (LG5). For the paternal linkage map, 91 markers were mapped from all three pseudo-testcross segregation types (Table 2, Figure 4). The length of the paternal linkage map was 420.8 cM, with the longest linkage group, LG5 (53.3 cM), and the shortest linkage group, LG8 (40.6 cM). The average interval marker distance was 5.2 cM and the maximum gap size ranged from 3.1 cM (LG1) to 11.3 cM (LG3). Owing to this unambiguous assignment of linkage groups to chromosomes, the linkage groups were designated with the chromosome numbers (e.g., chromosome 1 = LG 1 etc.).

### 3.3. Regeneration Rate from Microspore Culture

The microspore cultures were repeated twice. After completing the microspore culture experiment, each embryonic body was induced for about a month, and the induced embryos were confirmed. The average number of embryos was 3.52 in 62 F1 individuals. Only embryos that were not contaminated were selected and cultured on the MS medium. The average number of embryos cultured on the MS medium was 1.71 (Table 3). The regeneration rate was calculated using the formulas shown in the Materials and Methods. As we can see from the averages, the overall regeneration rate was quite low. The individuals without any embryogenesis totaled 31 of the F1 individuals.

### 3.4. QTL Mapping Analysis of Regeneration Rate and the Candidate Genes Related to Microspore Culture

**Figure 5:**
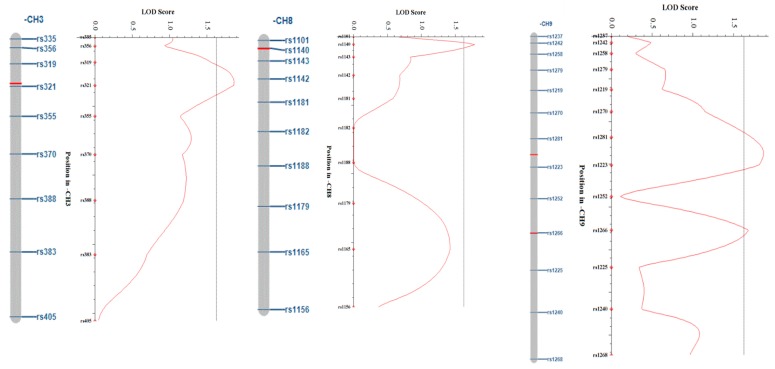
Logarithm of odds (LOD) score plots for chromosomes containing QTL with LOD scores (GX50). The horizontal lines indicate the thresholds for the LOD.

**Figure 6:**
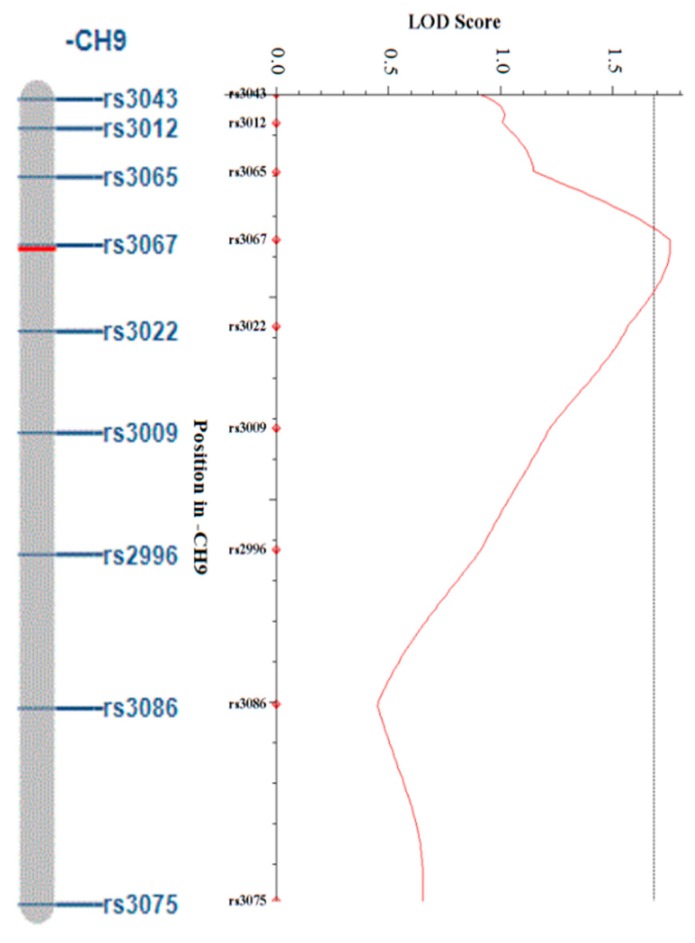
LOD score plots for chromosome 9 containing QTL with LOD score of 1.75 (GX71). The horizontal lines indicate the thresholds for the LOD.

Using SNP markers obtained through the GBS analysis, linkage maps of 439.2 cM and 420.8 cM, respectively, were constructed, and a QTL analysis was performed by ICIM (inclusive composite interval mapping) by inputting individual phenotypic values corresponding to traits related to the regeneration rate. For the QTL peak of the trait, only those above the threshold value of the trait were considered as significant peaks. From the results of the QTL analysis, four QTL peaks in GX50 were observed (one in chromosome 3, one in chromosome 8, and two in chromosome 9). The LOD values for each QTL are 1.86, 1.79, 1.87, and 1.68, respectively. The additive effect appears to be negative, indicating that those alleles originate from GX71. The phenotypic variations of peaks were 5.81%, 5.54%, 6.25%, and 5.24%, respectively, and all the values are within a 5–7% range and represent small values (Table 4, Figure 5). In the QTL analysis of GX71, only one QTL peak was observed in chromosome 9. The LOD value is 1.75 and the additive effect is positive, indicating that the allele also originates from GX71. In addition, the phenotypic variation was 13.19%, which was somewhat higher than the GX50 (Table 5, Figure 6). A candidate gene approach was used to determine whether specific loci may explain the responses for the regeneration rate observed in two lines selected for a high regeneration rate and a low regeneration rate. At the position of each QTL, we looked up genes corresponding to 1Mb up and down stream sequences based on left and right markers. Genes were identified in the Radish Genome Database (http://radish-genome.org/), and candidate genes for regeneration were selected based on previous studies (Table 6).

## 4. Discussion

### 4.1. Phenotypic Variation of Regeneration Rate in Parent Lines and F1 Population

The radish is a highly self-incompatible plant, and it is difficult to produce homozygous lines due to this characteristic. In previous studies, the parental lines with fixed traits through generation advancement were used for QTL analysis [34,35]. In cross-fertilization plants, bud pollination should be done by opening immature pollen and attaching pollen to mature flowers. Accordingly, it takes a lot of time and effort to develop lines with any traits fixed. In this study, in order to resolve this problem, a haploid breeding method has been attempted to obtain homozygous plants in a short period of time by doubling chromosomes [14,15]. Since the QTL analysis is based on allelic differences between parent lines, it is important to select parents that exhibit a broad range of phenotypic variations for the trait. Based on the comprehensive judgment, GX50 and GX71 were selected for crossing to generate an F1 mapping population, showing differences in terms of regeneration rate. The experiments showed that over 50% of the population did not regenerate, and only a few have high regeneration rates. These regeneration rates are consistent with previous findings [23,36].

### 4.2. GBS Analysis Using an NGS Platform and Genetic Mapping Using SNP Markers

This study developed markers showing polymorphism between two parental lines, and genotyping for all F1 individuals was completed for a number of polymorphic markers. An next-generation sequencing (NGS) analysis is a sequencing method that can generate large amounts of genomic information at a low cost and is applied for genomic research of various plants. Unlike the costly and time-consuming re-sequencing, GBS analysis is a technique that uses a reduced representation library (RRL) to partially sequence a genome of interest using restriction enzymes [37]. Since GBS does not sequence the entire genome, it can reduce the complexity of the chromosome to allow a complex genome analysis, and large numbers of SNPs can be found for a variety of plant genetic studies, including genetic mapping and population analysis [23,38]. Creating a linkage map plays a very important role in detecting the location of loci in the chromosome, such as QTL analysis. SNPs are the most frequently occurring genomic variations and can be used as markers for genome analysis, mapping, marker-assisted breeding, etc. The GBS method was adopted to obtain a large amount of SNPs in this study. In the preparation of the GBS library, a double-digestion method using *Nsi*l-HF and *MseI* was applied to reduce the genomic representation so that the sequence coverage for each allele increases. The library was generated with an average size of 344 bp. Considering that the size of the library suitable for NGS analysis is 170 ~ 350 bp, the generated library is considered suitable for sequencing [37]. Through the high filtering, 4462 high quality SNP markers were extracted and filtered again to generate 439.2 cM and 420.8 cM linkage maps using 178 markers, respectively. The average distance of the markers is 5.9 and 5.2 cM, respectively. When considering the length of the entire map, the interval between the markers is decent, but the length per linkage group is short [39,40]. The short linkage groups could be caused by two reasons: (1) the number of F1 individuals is small, and (2) we excluded redundant SNPs (could be reside in the same LD blocks). The genetic maps generated by the GBS have common lengthy linkage groups because some redundant markers overestimate the marker intervals. In this study, we tried to avoid those overestimated intervals by removing redundant SNPs. However, the number of markers mapped may be sufficient for a downstream analysis since we excluded duplicated markers in the same LD blocks. In addition, the SNP markers were mapped based on the locations in the pseudomolecule-level reference genome, and the physical position represented by the marker is clear, which may be useful for QTL mapping and maker-assisted selection (MAS).

### 4.3. QTLs Associated with Regeneration Rate

Candidate gene approaches have emerged as a way to merge QTL analysis with extensive data on traits involved in the regeneration rate. Plant regeneration is an important step in the success of a plant improvement program using tissue culture technology. The regeneration of plants is achieved through embryogenesis or tissue development [41]. The results of QTL analyses on plant regeneration rate were reported for several crops, such as rice [42,43], wheat [44], and barley [45,46,47], and a QTL analysis on plant regeneration rate was also reported in *B. oleracea* [48]. However, most of these or other plants are the results of QTL analyses involved in regeneration rates via anther culture [49,50] or tissue culture [51,52,53,54]. The QTL analysis of the regeneration rate in *Raphanus sativus* has not been reported to date, but a single marker analysis to find candidate genes involved in the efficiency of microspore culture has recently been reported [23]. There have been many reports on experiments on heat treatment, plant growth regulators, temperature controls, and light conditions to find suitable culture conditions for microspore culture for the regeneration of plants in Brassicaceae [55,56,57,58,59,60]. In the present paper, based on a review paper published by Ikeuchi [61], the genes related to regeneration were identified as candidate genes. The genes Rs426380 and Rs426400 have the same function as AT4G32540, which is known to function as the flavin-binding monooxygenase family protein and is known to act as an enzyme in auxin biosynthesis [62]. It also plays a key role in *de novo* root formation [63,64]. Auxin biosynthesis makes a contribution to root regeneration from leaf explants as well, because taking root is repressed by the chemical inhibition of auxin biosynthesis or YUC1, YUC2, YUC4, and YUC6 quadruple mutants that are faulty in auxin production [65]. The Rs465100 gene has the same function as AT5G51230, which is known as the PRC2 subunit of VEFS-Box of polycomb protein. In PRC2 mutants, these PRC2-targeted genes are ectopically expressed, resulting in spontaneous somatic dedifferentiation, callus formation, and embryonic development [66]. The genes Rs479580 and Rs479680 have the same functions as Arabidopsis’s AT4G02020, which is known as a SET domain-containing protein, also known as the PRC2 subunit. PRC2, one of the polycomb-group proteins, has histone methyltransferase activity and primarily trimethylates histone H3 on lysine 27 (H3K27me3). In Arabidopsis, PRC2 is known to inhibit the process of allowing embryos to mature during plant development [67].

## 5. Conclusions

In this study, a genetic map related to regeneration rate was developed through microspore culture. The genetic map used a large number of SNP markers generated using the GBS analysis method. Due to the F1 mapping, a maternal linkage map and a paternal linkage map are created separately and contain nine LGs. The phenotype was determined by the regeneration rate of embryos resulting from the microspore culture. QTL analysis was performed from an F1 population created by a cross between high and low regeneration varieties of radish by combining the genetic map with phenotypic data. Among the radish genes in the QTL region, genes known to be related to plant regeneration were designated as candidates. We found a total of five QTLs and predicted five candidate genes. These candidate genes are divided into two classes and appear to be either PRC2 subunits or auxin synthesis. Although the detected QTL information should be confirmed through repeated experiments, it may be useful for genetic research and MAS by developing molecular markers in the near future. Based on these results, it is expected that it will help to establish a system to proactively test the efficiency of the microspore culture. In addition, we are planning to develop PCR-based molecular markers associated with microspore culture so that breeders can speed up selecting radish lines with easier haploid generations. Moreover, we expect that the candidate genes will be further investigated in the near future with reverse genetics approaches to find out exact functions related to microspore culture.

## Figures and Tables

**Table 1:** Length, number of markers, average spacing, and largest intervals of GX50 genetic maps (LG; linkage group, SNP; single nucleotide polymorphism, cM; centi-Morgan).

LGs	Length (cM)	Number of SNPs	Average Interval (cM)	Largest Interval (cM)
1	40.2	7	6.7	8.8
2	36.7	7	6.1	7.9
3	47.9	9	6.0	8.5
4	55.2	10	6.1	10.8
5	56.0	13	4.7	13.4
6	55.2	11	5.5	10.9
7	51.7	7	8.6	11.8
8	42.2	10	4.7	8.9
9	54.1	13	4.5	7.7
Total	439.2	87	5.9	

**Table 2:** Length, number of markers, average spacing, and largest intervals of GX71 genetic maps.

LGs	Length (cM)	Number of SNPs	Average Interval (cM)	Largest Interval (cM)
1	41.4	8	5.9	3.1
2	47.9	11	4.8	6.7
3	50.7	9	6.3	11.3
4	51.8	10	5.8	10.8
5	53.3	11	4.2	11
6	44.8	13	3.7	7.8
7	41.8	13	3.5	6.7
8	40.6	7	6.8	8.3
9	48.5	9	6.0	10.4
Total	420.8	91	5.2	

**Table 3:** The average number of embryos and cultures of the F1 individuals.

	F1 Individuals
Total embryo average ^1^	3.52 ^3^
Total culture average ^2^	1.71

^1^ The average number of embryos from the entire 62 F1 individuals with two replications. ^2^ The average number of culturable embryos (not contaminated) from the total amount of embryos. ^3^ Rounded to two decimal places.

**Table 4:** Effects of the QTLs associated with the regeneration rate detected in F1 populations (GX50).

QTL	LOD	A.E^1^	PVE(%) ^2^
LG3_1	1.86	−6.13	5.81
LG8_1	1.79	−5.96	5.54
LG9_1	1.87	−6.38	6.25
LG9_2	1.68	−5.74	5.24

^1^ Estimated additive effect of QTL. Negative values indicate effects from GX71; Positive values indicate effects from GX50. ^2^ Phenotypic variation explained by QTL.

**Table 5:** Effects of the QTL on the regeneration rate detected in F1 populations (GX71).

QTL	LOD	A.E ^1^	PVE(%) ^2^
LG9_1	1.75	5.90	13.19

^1^ Estimated additive effect of QTL. Negative values indicate effects from GX50; Positive values indicate effects from GX71. ^2^ Phenotypic variation explained by QTL.

**Table 6:** List of *Arabidopsis* orthologs associated with plant regeneration near the QTL region.

QTL	Gene ID	A.T ortholog	Gene Description
P1_Chr8_1^1^	Rs426380	AT4G32540	Flavin-binding monooxygenase family protein
P1_Chr8_1	Rs426400	AT4G32540	Flavin-binding monooxygenase family protein
P1_Chr9_1/P1_Chr9_2	Rs465100	AT5G51230	VEFS-Box of polycomb protein
P2_Chr9_1^2^	Rs479580	AT4G02020	SET domain-containing protein
P2_Chr9_1	Rs479680	AT4G02020	SET domain-containing protein

^1^ P1 is parent 1, meaning GX50. ^2^ P2 is parent 2, meaning GX71.
